# Chromosome architecture as a determinant for biosynthetic diversity in Micromonospora

**DOI:** 10.1099/mgen.0.001313

**Published:** 2024-11-05

**Authors:** David R. Mark, Nicholas P. Tucker, Paul R. Herron

**Affiliations:** 1 Strathclyde Institute of Pharmacy and Biomedical Sciences, University of Strathclyde, Glasgow, G4 0RE, UK; 2 School of Infection and Immunity, University of Glasgow, Glasgow, G12 8TA, UK; 3 School of Allied Health Sciences, University of Suffolk, Ipswich, IP3 0FS, UK

**Keywords:** chromosome biology, genomics, *Micromonospora*, natural products

## Abstract

Natural products – small molecules generated by organisms to facilitate ecological interactions – are of great importance to society and are used as antibacterial, antiviral, antifungal and anticancer drugs. However, the role and evolution of these molecules and the fitness benefits they provide to their hosts in their natural habitat remain an outstanding question. In bacteria, the genes that encode the biosynthetic proteins that generate these molecules are organised into discrete loci termed biosynthetic gene clusters (BGCs). In this work, we asked the following question: How are biosynthetic gene clusters organised at the chromosomal level? We sought to answer this using publicly available high-quality assemblies of *Micromonospora*, an actinomycete genus with members responsible for biosynthesizing notable natural products, such as gentamicin and calicheamicin. By orienting the *Micromonospora* chromosome around the origin of replication, we demonstrated that *Micromonospora* has a conserved origin-proximal region, which becomes progressively more disordered towards the antipodes of the origin. We then demonstrated through genome mining of these organisms that the conserved origin-proximal region and the origin-distal region of *Micromonospora* have distinct populations of BGCs and, in this regard, parallel the organization of *Streptomyces*, which possesses linear chromosomes. Specifically, the origin-proximal region contains highly syntenous, conserved BGCs predicted to biosynthesize terpenes and a type III polyketide synthase. In contrast, the ori-distal region contains a highly diverse population of BGCs, with many BGCs belonging to unique gene cluster families. These data highlight that genomic plasticity in *Micromonospora* is locus-specific, and highlight the importance of using high-quality genome assemblies for natural product discovery and guide future natural product discovery by highlighting that biosynthetic novelty may be enriched in specific chromosomal neighbourhoods.

**Impact Statement**

Publicly available genome data represent a rich source of information for the discovery of natural products. Here, we leverage these data to demonstrate the chromosome level of biosynthetic gene clusters in *Micromonospora*. We demonstrate that the BGCs in the origin-proximal region of the chromosome are conserved, whereas the origin-distal region BGCs are not. Finally, we postulate that this may reflect these organisms’ ecology, whereby ‘more useful’ BGCs accumulate in the chromosomal core, whereas situationally useful BGCs only transiently occupy the chromosome, occupying a region with high genetic turnover.

## Data Summary

Assemblies used for the analyses described herein are listed in Table S1.

## Introduction

Surpassing fungi and other bacteria in their ability to biosynthesize societally useful natural products [1], the actinomycetes – hyphal, spore-forming members of the phylum *Actinomycetota* – account for the production of many clinically used antibiotics as well as anticancer drugs and contributed hugely to the golden age of antibiotic discovery [2]. However, these natural products are not produced for human benefit and have evolved over millions of years to provide fitness benefits to the organisms that host them through various functions, such as contributions to interspecies interactions [3,5] and tolerance of adverse environmental conditions [6,7]. The chemical space occupied by natural products is broad and diverse, encompassing a large variety of potential molecules, including polyketides [8], ribosomally synthesised [9] and nonribosomally synthesised [10] peptides, aminoglycosides [11,13] and modified nucleosides [14,15].

Control of the metabolically expensive biosynthesis of these molecules is maintained through a suite of regulatory mechanisms, ensuring that these metabolites are only synthesised as they are needed, such as coinciding with developmental or environmental cues [16,18]. Genes encoding these control mechanisms are often located adjacent to genes encoding the biosynthesis of specialised metabolites as well as genes encoding other functions, such as resistance to toxic metabolites and machinery for their transport. These regions are termed ‘biosynthetic gene clusters’ (BGCs) [19]. A large proportion of genomic space is often dedicated to housing BGCs, approaching 10% of the total genome content in some organisms, such as *Salinispora* [20]. However, how this ‘genomic real estate’ is allocated is an ongoing subject of interest.

We chose to investigate this question by examining the actinomycete genus *Micromonospora*. Despite being second to *Streptomyces* for the number of antibiotics they produce [3], their biology in terms of development and physiology is still poorly understood. Taxonomic and phylogenetic analyses of *Micromonospora* suggest that the genus is difficult to resolve from closely related genera, such as *Xiangella*, *Salinispora* and *Verrucosispora*, and indeed suggest that some ‘*Micromonospora*’ may be misclassified [21]. The genus was once thought to possess a linear chromosome, similar to that of *Streptomyces,* following pulsed-field gel electrophoresis [22]; however, whole-genome sequencing of isolates showed that they possess circular chromosomes instead [23,24]. There have been no studies on the initiation and termination of DNA replication in this genus. In addition to this, both plasmids [23,25] and phage have been isolated from members of the genus [26]. They begin their life cycle as a spore, which germinates to form a vegetative mycelial mat growing into the substrate medium, with one of the characteristic features of *Micromonospora* being the formation of individual monospores to enable dispersal [3]. Although there have been few studies on *Micromonospora* regarding growth and development, their proclivity for natural product production means that there is a wealth of genome sequences available for analysis [27]. As of 2022 [28], there are 225 *Micromonospora* RefSeq assemblies available from the NCBI [29]. This enables easy placement of novel strains in their taxonomic context and allows for comparisons to be made between strains’ abilities to produce natural products.

Work has been done to understand the composition of the *Micromonospora* core and pangenome – the genes shared between the members of a set of *Micromonospora* and unique to members of that set, respectively [30]. The industrial utility and clinical significance of actinomycetes have also driven interest in how they organize the BGCs involved in the production of their natural products. Studies on *Micromonospora* have largely focussed on their capacity for making bioactive molecules [31]. However, the composition and organization of their circular chromosomes in comparison with the linear chromosomes of streptomycetes are unknown. What we do know about *Streptomyces* is that their linear chromosomes are marked by a central core containing the origin of replication, flanked by two arms where BGCs tend to accumulate [32,33]. The core of *Streptomyces* chromosomes serves as an axis around which recombination occurs [34]. This pattern has been mirrored in *Amycolatopsis* [35] and *Salinispora* [36] chromosomes, although these observations were made from short-read sequenced genomes that may have introduced bias in BGC prediction and analysis [37]. Work in better-studied organisms has identified molecular drivers of chromosome architecture, such as the FtsK-orienting polar sequences involved in replication termination [38] and architecture-imparting sequences that restrict horizontal gene transfer [39].

At the time of data collection for this work, there were 30 assemblies of *Micromonospora* available onNCBI (https://www.ncbi.nlm.nih.gov/), which were either complete or chromosome level [29]. These assemblies present a dataset that can help us understand the organization and evolution of these organisms. Here, we examined the organization of BGCs within high-quality assemblies, first by looking at the larger architecture of the chromosomes – identifying a conserved origin island with highly divergent regions distal to *oriC*. In addition, we aim to compare the location of BGCs on the circular chromosomes of *Micromonospora* with those on the linear chromosomes of *Streptomyces*.

## Methods

### Curation of single-contig *Micromonospora* assemblies

To understand the evolution of specialised metabolism in *Micromonospora*, we chose to investigate the relative locations of BCGs in *Micromonospora* chromosomes. To enable this, we first sought to collate a set of single-contig assemblies of *Micromonospora* by downloading NCBI RefSeq assemblies with completion levels of either ‘complete’ or ‘chromosome’. As the DNA helicase-encoding *dnaA* is reliably located proximal to *oriC*, we decided to use this as a reference point for our analyses. Thus, we oriented these assemblies using SnapGene [40] so that they started at the first base of *dnaA* to compare the locations of BGCs. To identify large misassemblies in our dataset, we performed an all-vs-all comparison using Nucmer, implemented in mummer4 v. 4.0.0rc1 [41], followed by the show-coords command to highlight alignment location. This led us to discard *M.* sp. L5 and *M.* sp. B006 based on these alignments, as they contained what we believe to be large misassembly errors (Fig. S2, available in the online version of this article). The raw assembly data used for analysis were obtained on 24 May 2021. Closed streptomycete chromosome sequences, flanked by telomeric sequences at both ends, were used to compare the location of BGCs between the linear and circular chromosomes of *Micromonospora*.

### DNA strand coding bias

We investigated whether *Micromonospora* preferentially encodes genes on the leading or lagging strands of its chromosome. To achieve this, we reoriented the assemblies using Prokka (Galaxy Ver. 1.14.6) to obtain GFF3 format files. The files were imported using the read.gff function in ape (ver. 5.6–2) [42] and filtered for genes, with their position normalised using the same calculation applied for BGCs. GFF3 format files assign ‘+’ or ‘−’ to indicate if genes are on the top or bottom strand of a DNA molecule – to convert this to leading or lagging, we considered bottom strand genes on the left replichore (upstream of *dnaA* to the mid-chromosome) to be leading and top strand genes to be lagging, with the opposite rules applied to the left replichore. We compared strand bias per organism that we tested using a paired t-test.

### ANI calculation of complete *Micromonospora* assemblies

To understand the relative relatedness of the complete *Micromonospora* assemblies in our analysis as identified by autoMLST, we generated an all-vs-all average nucleotide identity (ANI) comparison using FastANI (Galaxy Version 1.3). The values generated by this were used to generate a heatmap using the gplots package (ver. 3.1.3) [43] in R.

### BGC locus mapping

To map the loci of BGCs present in the dataset of single-contig assemblies, we utilised antiSMASH 6.0 [44]. Sequence data from our dataset were exported in the FASTA format and uploaded to the antiSMASH web server, with BGCs being predicted under the antiSMASH ‘strict’ parameters to reduce the risk of false-positive BGCs being identified. In addition, runs were carried out with ‘ClusterBlast’, ‘SubClusterBlast’, ‘MIBiG cluster comparison’, ‘ActiveSiteFinder’, ‘RREFinder’, ‘Cluster PFAM analysis’, ‘Pfam-based GO term annotation’ and ‘TIGRFam analysis’ enabled.

Once the BGCs present in our assemblies were identified, we then calculated the midpoint of the BGCs with the following formula: $B_{M}=\frac{B_{1}+B_{2}}{2}$, where $B_{M}$ is the midpoint of a given BGC, $B_{1}$ is the position of the first nucleotide in the BGC and $B_{2}$ is the position of the last nucleotide in the BGC. To control for variation in chromosome size, we then normalised $B_{M}$ on a scale of 0–100 using the following formula: $NB_{M}=100(\frac{B_{M}}{S})$, where ${NB}_{M}$ is the normalised midpoint of a given cluster and S is the size in base pairs of the chromosome the BGC occupies.

### Between-cluster comparisons

To test if BGCs of the same type at similar *loci* between chromosomes were homologous, we utilised BiG-SCAPE (v. 1.1.5) [45] and Clinker (v.0.0.27) [46], both using default parameters. BiG-SCAPE enabled us to generate networks of related BGCs and Clinker used to generate alignments of syntenous BGCs to investigate their gene content. These networks were further investigated using Cytoscape software (v. 3.7.1). Clinker was used to generate pairwise alignments between BGCs present in a network, which were coloured according to their functions predicted by antiSMASH.

### Ecological modelling of *Micromonospora* BGCs

To test if there was a difference in the diversity of BGCs present in either half of the *Micromonospora* chromosome, the BGCs in our dataset were split into two sets: ‘origin-proximal’, i.e. a quarter chromosome upstream and downstream of *dnaA* or ‘mid-chromosome’, i.e. a quarter chromosome upstream and downstream of the mid-chromosome. These were analysed by BiG-SCAPE, and the resulting presence–absence tables were then used to calculate the Shannon, Simpson and inverse Simpson diversity indices using the vegan package [47] (ver. 2.6-2) in R.

### R version

This work was carried out on multiple R versions, starting from v. 3.6.2 – the code used for analysis and plotting has been validated as functional in R v 4.3.1.

## Results

### Preparation of a dataset of well-assembled *Micromonospora* chromosomes

**Fig. 1.**
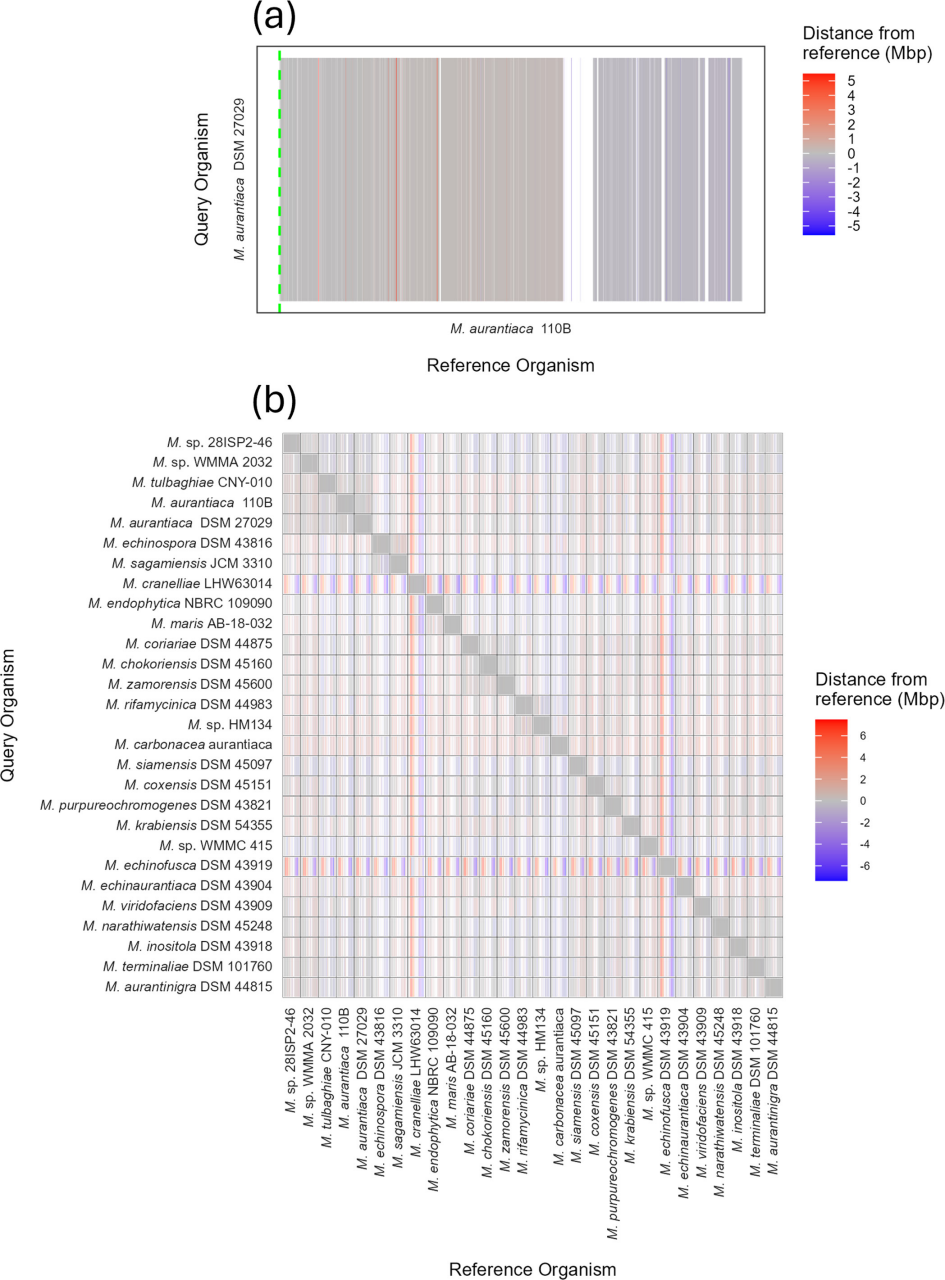
Conservation of the origin-proximal region of *Micromonospora* chromosomes. Pairwise whole-genome alignments of *Micromonospora* generated using Nucmer. (**a**) Illustrative output of *M. aurantiaca* DSM 27029 aligned to *M. aurantiaca* 110B, both oriented to begin at *dnaA* (hatched green line). Grey bars indicate syntenous regions – regions that align and are at the same position on the chromosome – and white gaps indicate regions of the chromosome to which nothing aligned. Extrapolated to an all-vs-all comparison of 28 strains. (**b**) These alignments highlight that the origin-proximal region (left and right edges of the panels) is conserved, whereas the mid-chromosome (centre of the panels) contains large gaps.

Our dataset began with 30 *Micromonospora* chromosomes, covering complete and chromosome-level assemblies available on the NCBI. We began by using Nucmer to generate all-vs-all pairwise alignments to identify misassemblies present in this set, after which we discarded *Micromonospora* sp. B006 and *Micromonospora* sp. L5 (Fig. S2), reducing the number of assemblies to 28. We discarded *Micromonospora* sp. B006 because its alignments suggested either misassembly or multiple chromosomal rearrangements that were absent in the other assemblies. *Micromonospora* sp. L5 was discarded owing to a large inversion in its assembly, which, if true, would bring the mid-chromosome implausibly close to the origin of replication. Table S1 contains the members of our set and their Genbank accession numbers. In addition, the Nucmer alignments indicated a conserved architecture in *Micromonospora* chromosomes, with high levels of synteny close to the chromosomal origin of replication contrasted by a less well-structured mid-chromosome (Fig. 1a, b).

Having discarded poorly assembled genomes from our set, we next aimed to ensure that all our assemblies belonged to the same reported genus. Using FastANI, we achieved this – the minimum ANI value was 82.713% between *M. chokoriensis* DSM 45160 and *M. echinospora* DSM 43816 and the maximum between two different assemblies was 98.903% between *M. aurantiaca* DSM 27029 and *M. aurantiaca* 110B (Fig. S1). This confirmed that all the members of our dataset belonged to *Micromonospora* [48].

### The *Micromonospora* chromosome has conserved architecture

**Fig. 2.**
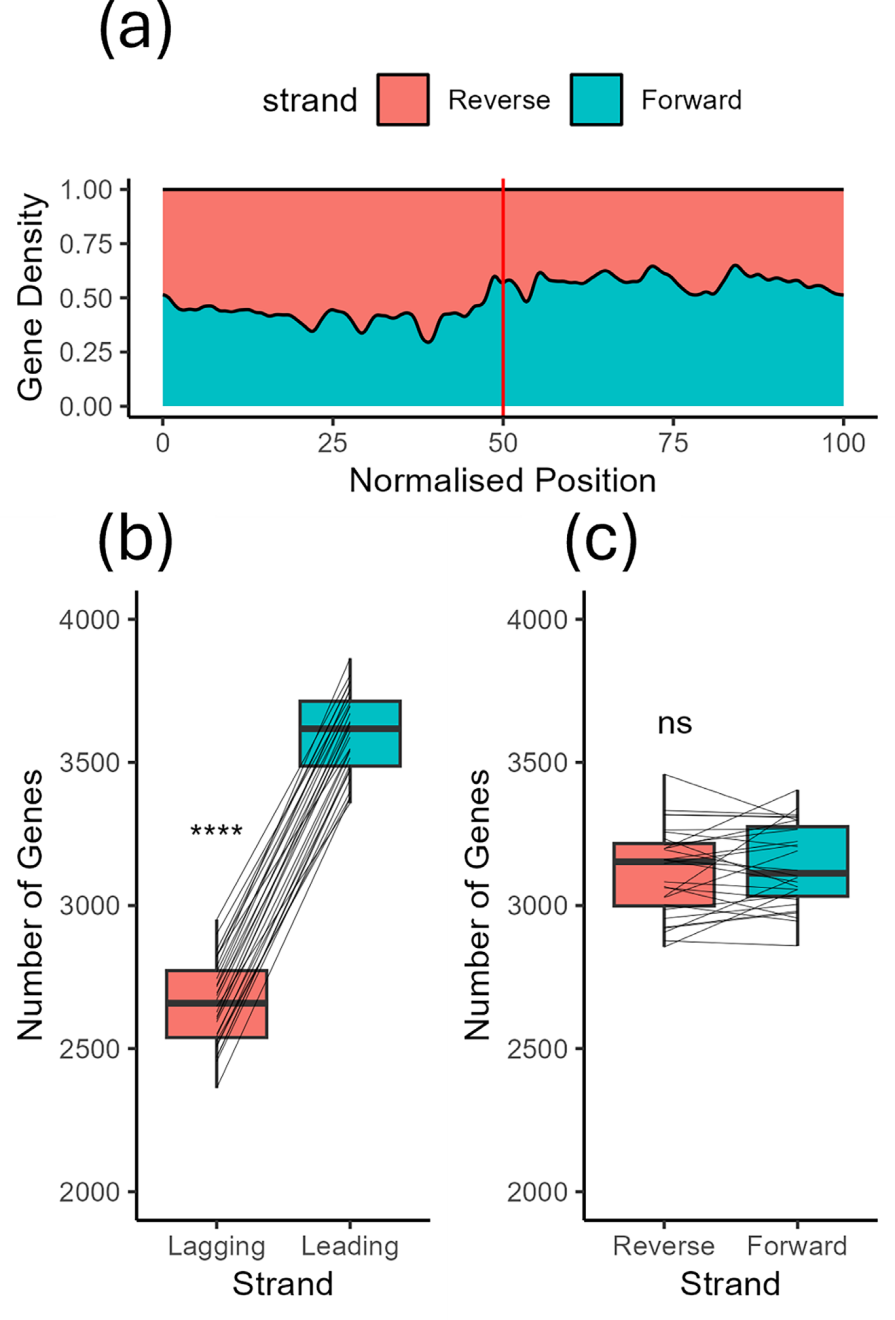
*Micromonospora* preferentially encodes genes on the leading strand instead of the lagging strand. (**a**) Kernel density estimate of gene bias along *Micromonospora* chromosomes. The vertical red line indicates the position of *dnaA*. Upstream of *dnaA*, genes are preferentially encoded on the reverse strand and vice versa. (**b**) and (**c**) Boxplots illustrating that *Micromonospora* preferentially encodes genes on the leading strand, with no preference for encoding to the forward or reverse strand. *P* values were calculated by t-test, ****P<0.001.

Upon examination of the alignments generated by Nucmer, we observed conserved chromosomal architecture. Specifically, the origin-proximal region of the chromosome is highly conserved, whereas the origin-distal region shows much less conservation (Fig. 1a, the alignments are available in high resolution in Fig. S3). This suggests that the terminus-proximal region of the *Micromonospora* chromosomes acts as a hotspot of recombination compared to the origin, similar to that found in *Streptomyces* [34]. We also observed inversions in the origin islands of *M. cranelliae* LHW63014 and *M. echinaurantiaca* DSM 43904. These large inversion events led us to ask what role gene-strand bias plays in *Micromonospora* chromosome architecture and if the leading or lagging strand of DNA was enriched for coding sequences. We asked this as, hypothetically, the inversions in *M. cranelliae* and *M. echinaurantiaca* concomitantly invert any strand–gene content relationship. The *Micromonospora* strains in our dataset have significantly more genes on the leading strand of either replichore, including the two strains with inverted origin islands (Fig. 2b); however, there was no difference between the number of genes on the top and bottom strands of DNA (Fig. 2c). This suggests that selection drives enrichment for genes on the leading strand of chromosomal DNA.

### *Micromonospora* possesses a rich and diverse repertoire of BGCs

**Fig. 3.**
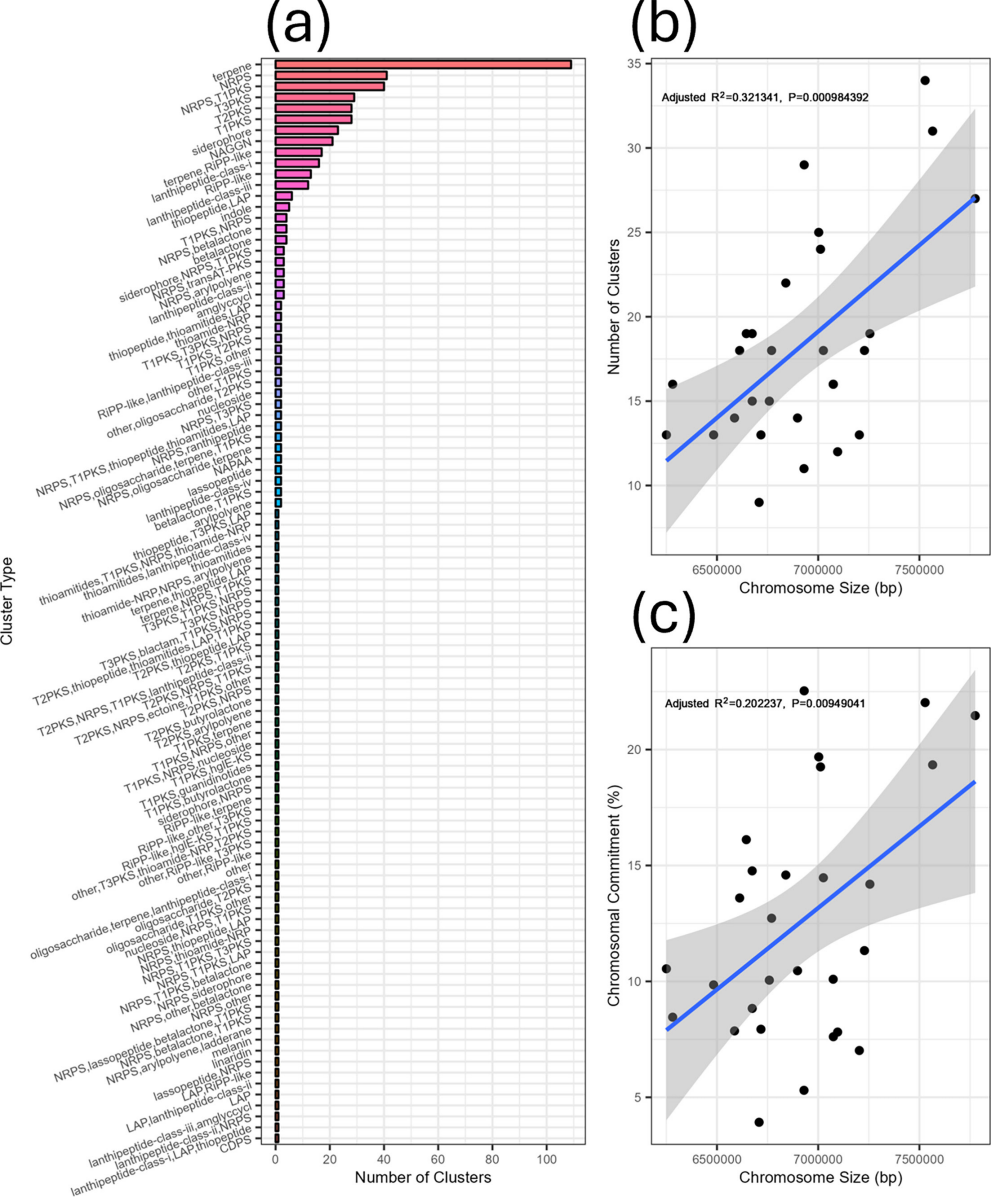
BGC accumulation partially drives chromosome growth in *Micromonospora.* (**a**) Number of BGCs of each antiSMASH type contained within our analysed chromosomes. While a few BGC types – such as terpenes and NAGGN clusters – were well represented, there were also many cluster types with only a single representative. (**b**) Correlation of the number of encoded BGCs in each *Micromonospora* chromosome against chromosome size. (**c**) Linear regression of the percentage of the *Micromonospora* chromosome committed to hosting BGCs and chromosome size.

Knowing that our dataset was populated only by fully assembled *Micromonospora* chromosomes, we then passed them onto antiSMASH to elucidate the number and nature of BGCs possessed by them. This revealed that the genus is rich in BGCs, possessing 99 different types between them and a total of 511 BGCs, dominated by terpene, PKS and NRPS classes (Fig. 3a). The mean chromosome size in the assemblies was 6914 258 bp, and the mean number of BGCs carried was 18.25 with wide variation between organisms. After noting the variability between *Micromonospora* chromosome sizes and the number of BGCs they possessed, we sought to see if the two values were correlated. To do this, we performed a linear regression between the number of BGCs present in our organisms and the size of their chromosomes (Fig. 3b). Understanding that BGCs vary in size, we also sought to see if there was a correlation between the percentage of the chromosome occupied by BGCs and chromosome size (Fig. 3c). We found a weak but statistically significant positive correlation for both cases (*R*^2^=0.32, *P*<0.001 for number of clusters vs genome size; *R*^2^=0.20, *P*<0.001 for % commitment vs genome size), suggesting that BGCs both contribute to genome growth in *Micromonospora* and that larger chromosomes have more genomic space devoted to secondary metabolism. In addition, the proportion of the chromosome occupied by BGCs was highly variable and ranged from <5% of DNA content to >20% (Fig. 3c).

### BGCs are present in both the core and variable regions of the chromosome

**Fig. 4.**
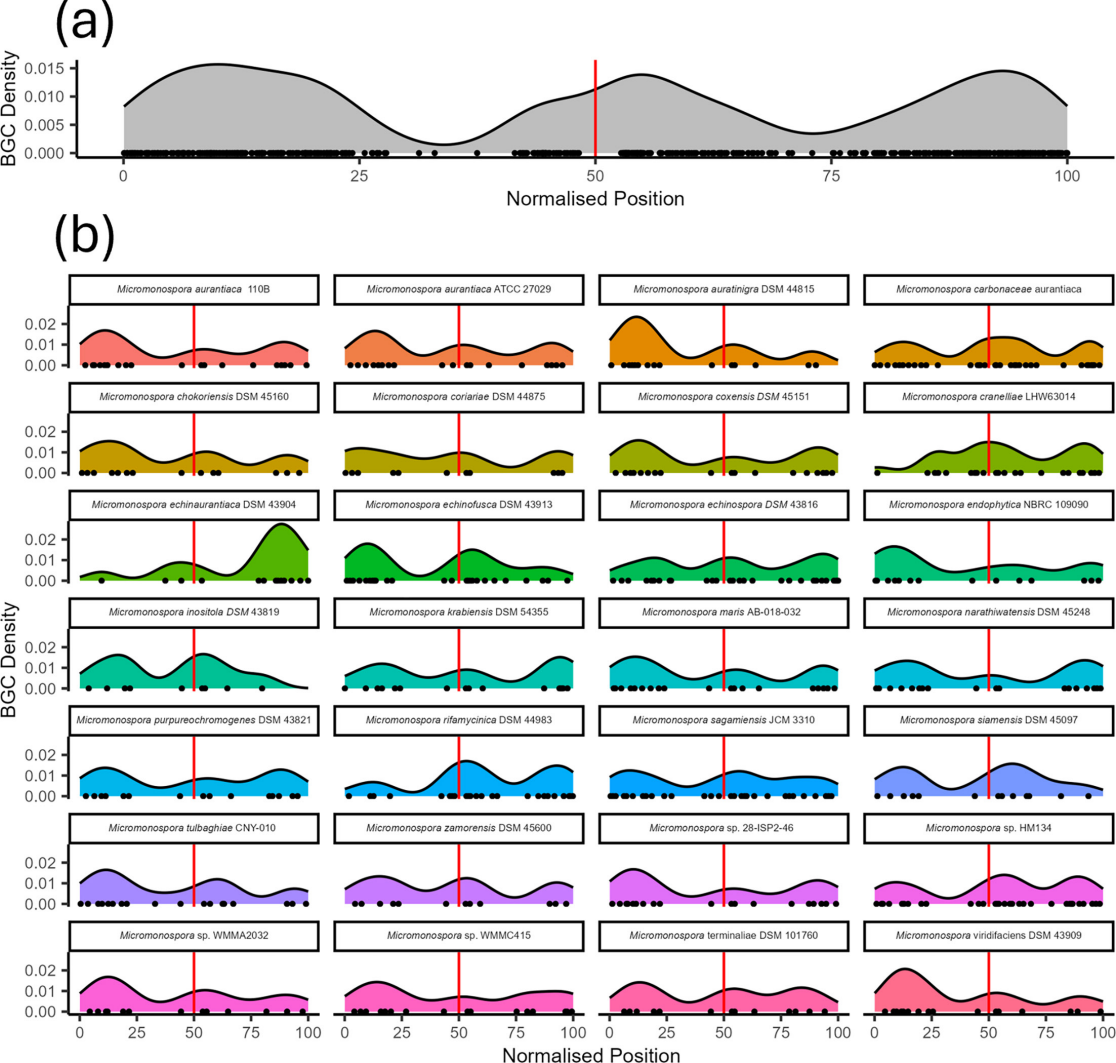
Micromonospora shows conserved BGC distribution. (**a**) Kernel density estimate of the chromosomal position of BGCs belonging to *Micromonospora*, with *dnaA* at position 50. (**b**) Kernel density estimates of BGC distribution by species. These indicate that *Micromonospora* have two BGC dense regions at opposite poles of their chromosome.

While performing quality control on our dataset, we observed that *Micromonospora* chromosomes have conserved architecture with syntenic regions close to the origin of replication and less synteny towards the mid-chromosome. This led us to compare the loci of BGCs in *Micromonospora* chromosomes. To achieve this, we normalised the loci of the BGCs and plotted them on a pseudochromosome. This revealed that there were two hotspots where BGCs accumulate at separate locations on *Micromonospora* chromosomes: one at the origin of replication and one at the terminus (Fig. 4a). This was the case for all 28 *Micromonospora* included in our dataset (Fig. 4b). From this, we can conclude that some chromosomal loci are favoured over others for BGC accumulation. Of note was that, at the organism level, BGC accumulation was favoured at one arm of the *ori*-distal region rather than being symmetrically distributed across the pole. Despite this, on average, there was no preference for the left or right arm at the generic level. We believe there are likely ‘hotspots’ for recombination, which are being driven by other factors on top of the distance from the origin of replication. Despite the difference in chromosome topology, we note that this distribution is analogous to that observed in the linear chromosomes of members of the *Streptomycetaceae* (Fig. S4).

### Homologous BGCs are syntenic across *Micromonospora* chromosomes

**Fig. 5.**
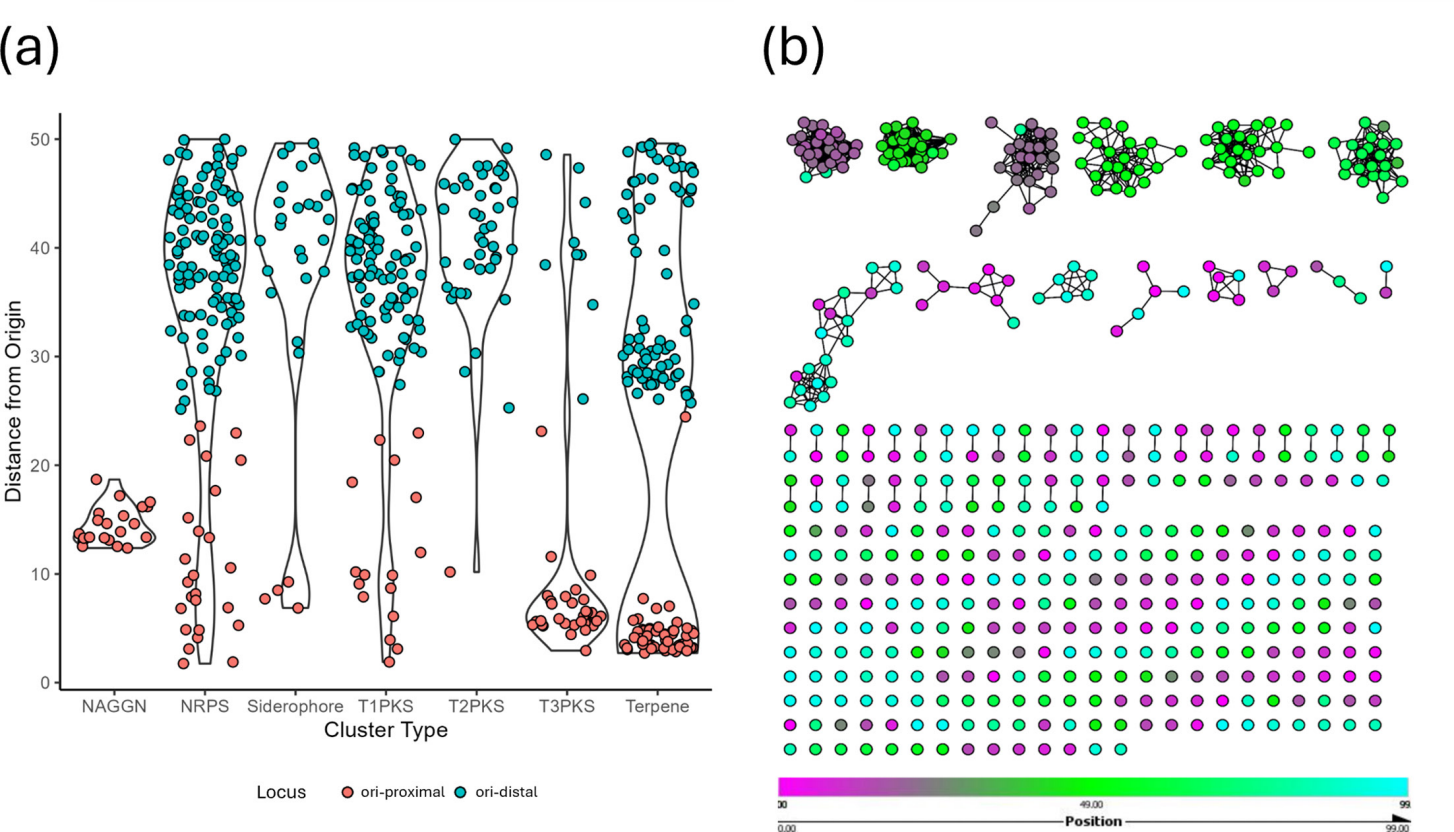
Homologous BGCs are syntenic in *Micromonospora*. (**a**) Violin plots of frequently occurring classes of BGC. NAGGN and T3PKS containing BGCs are notable as being present predominantly in the ori-proximal region (distance <25), whereas NRPS, T1/2 PKS and siderophore clusters are predominantly in the ori-distal region (distance >25). Terpenes are present in three groups in both the origin-proximal and origin-distal regions. (**b**) BiG-SCAPE network of BGCs coloured by the chromosomal position, with *dnaA* at position 50 (green) and the mid-chromosome at positions 0 and 99 (magenta and cyan). Homologous BGCs in our assemblies are syntenic and largely represented in the origin-proximal region. On the other hand, small gene cluster families and singletons were mostly located in the origin-distal region of the chromosome.

After showing that chromosomal regions are rich in BGCs, we then sought to examine the distribution of classes of BGCs across *Micromonospora* chromosomes. We found that some classes of BGC were enriched at origin-proximal chromosomal loci, whereas others were more common at the mid-chromosome (Fig. 5a). For example, T3PKS, terpene and NAGGN clusters were mostly located close to *oriC*, with NAGGN clusters having a median location at 14.5% (normalised distance) from the start of *dnaA* and T3PKS containing clusters at 6%. On the other hand, clusters containing NRPS, siderophore, T1PKS and T2PKS biosynthetic genes had median distances of 37.97%, 41.4%, 37.61% and 41.8%, respectively. Interestingly, terpene-containing clusters appeared to exist as three different populations – with one close to the origin of replication, one towards the mid-chromosome and one halfway between the two. From this, we concluded that BGC type affects where that cluster lies on the chromosome. However, the terpene clusters having three distinct populations demonstrate that this cannot be the only driver. Interestingly, deletion of the *oriC-*proximal Terp2 terpene cluster in *Salinispora* results in an apigmented phenotype owing to the deletion of precursor biosynthesis, whereas the disruption of the *oriC-*distal clusters only disrupts pigmentation due to the deletion of pigment-modifying enzymes [49]. Observing that there was a pattern of distribution where BGCs of given classes localised at particular regions of the *Micromonospora* chromosome, we hypothesised that these were in fact homologous BGCs. To test this, we generated a BiG-SCAPE network to group similar BGCs present in our dataset, annotated by the position of the BGCs on the chromosome. This network partially confirmed our hypothesis – BGCs at the origin of replication shared networks (Figs 5b and S5). We also observed that some BGCs were placed in networks of otherwise syntenic BGCs – these clusters belonged to organisms that Nucmer analysis suggested a historical inversion of the origin of replication and, thus, whether the BGC was located to the left or right of *dnaA*. We also observed that our dataset contained a large number of singleton BGCs, not associated with a network. These singletons mostly existed away from the origin of replication and thus we sought to see if the origin-proximal region of the chromosome and the mid-chromosome contained different populations of BGCs.

### *OriC*-distal BGCs show greater diversity than origin-proximal clusters

**Fig. 6.**
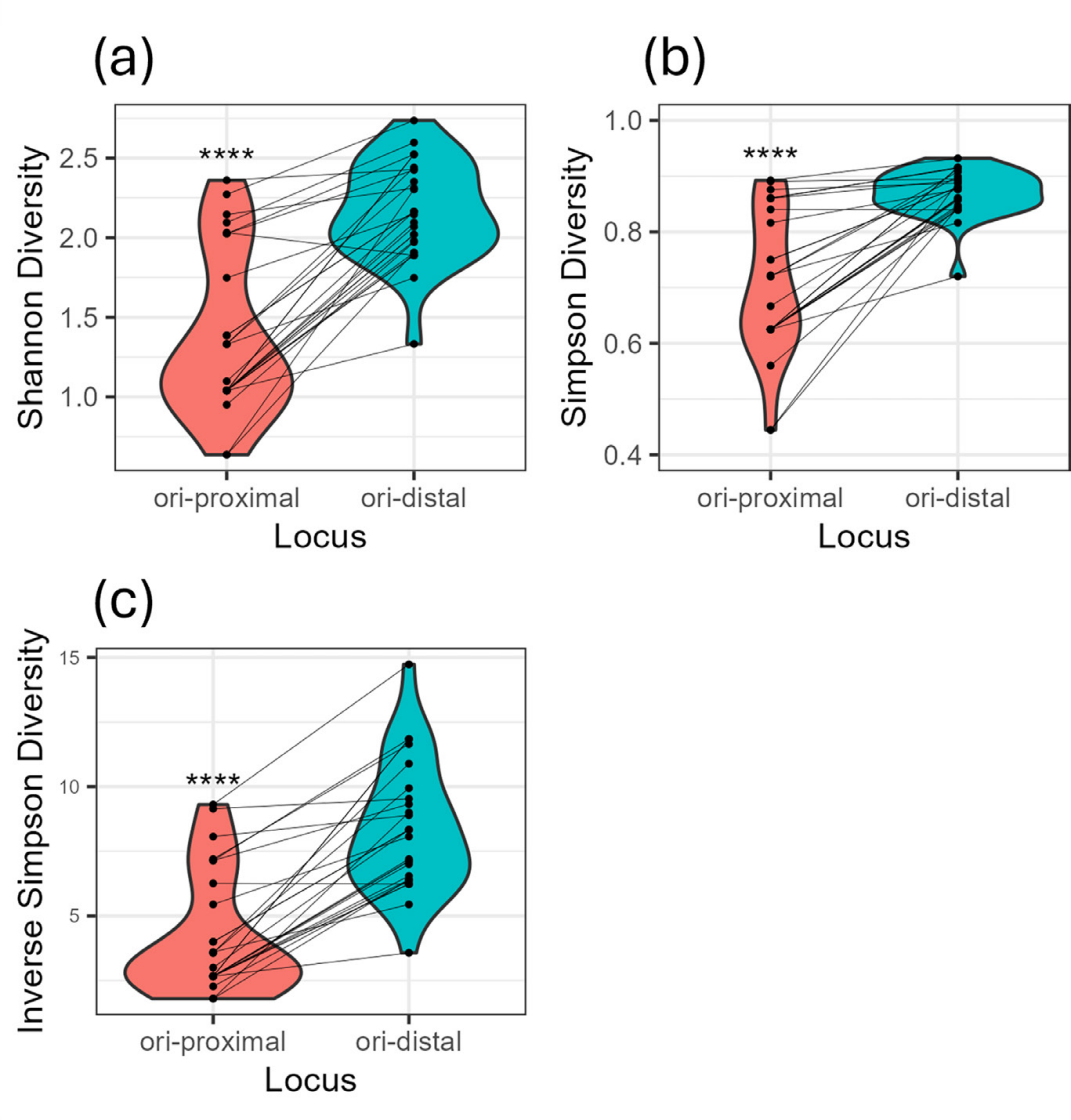
The ori-distal region of the *Micromonospora* chromosome contains a greater diversity of BGCs than the origin of replication. Shannon (**a**), Simpson (**b**) and inverse Simpson (**c**) diversities of the origin and mid-chromosome regions of *Micromonospora* chromosomes. For all measures, the mid-chromosome was more diverse than the origin. *P* values were calculated by Wilcoxon’s signed-rank test with continuity correction, ****P<0.001.

After noting that the largest networks of BGCs predominantly occurred close to the origin of replication, we hypothesised that BGCs close to the origin and those in the *oriC-*distal region could be described as distinct populations of BGC. To test this hypothesis, we divided the chromosome into two distinct regions: BGCs belonged to the origin region if their normalised locus was higher than 25 but less than 75, else they were designated as ori-distal BGCs. Using the gene cluster families identified by BiG-SCAPE in our previous network analysis, we showed that the origin region contained less BGC diversity than the mid-chromosome by Shannon, Simpson and inverse Simpson diversity indices (Fig. 6). This indicates that the ori-distal half of the chromosome has a more diverse BGC population than the origin of replication. The only strain exempt from this trend was * M. carbonaceae*, which incidentally encodes the greatest number of BGCs in our dataset.

## Discussion

The aim of the work described here was to characterize the genomics of secondary metabolism in *Micromonospora* by utilizing high-quality genome assemblies to support mapping the relative loci of BGCs within chromosomes. We first sought to collect a dataset of high-quality *Micromonospora* assemblies.

What is ‘high-quality’ is a subjective matter – here, we defined it as an assembly contained in a single contig whose predicted physical structure agreed with other published *Micromonospora* genomes. As *Actinomycete* genera contain conserved origin islands with a high degree of synteny [34], we believe that these criteria were sufficient to exclude large-scale, biologically implausible misassemblies from our dataset while not rejecting datasets based on events such as putative genome rearrangements. We chose to exclude *Micromonospora* sp. L5 owing to a rearrangement in its assembly which would unbalance DNA replication by bringing the terminus of replication in the mid-chromosome adjacent to the origin of replication. We chose to remove *Micromonospora* sp. B006 due to the large conserved regions that were asyntenic with other members of our dataset. As the positions of BGCs were calculated relative to the origin of replication of each organism, the calculated loci from these organisms would be spurious. ANI analysis supported that our members are all *Micromonospora*, with a minimum identity of 82.713% between organisms. This step was important to resolve the taxonomic identity of our organisms and ensure that they were related enough for comparing the loci of their BGCs to be worthwhile as well as by mitigating the possibility that their taxonomy had been misassigned [50].

The Nucmer alignments we performed as part of the quality control process also allowed insight into the architecture of *Micromonospora* chromosomes, revealing the ori-distal pole of the chromosome to be poorly conserved compared to the ori-proximal region, as well as highlighting the inverted origin islands of *M. cranelliae* and *M. echinaurantiaca*. We also examined our organisms for coding region strand bias, a frequently observed phenomenon across bacteria where genes are preferentially located on the leading strand of the chromosome [51]. In agreement with this, our organisms preferentially encoded genes on the leading strand of their chromosome. Upstream of *oriC*, genes were preferentially encoded on the minus strand of the chromosome, whereas downstream genes were preferentially encoded on the plus strand. This suggests that the mid-chromosome of *Micromonospora* also serves as the site of replication termination [52].

Our dataset enabled us to ask how committed *Micromonospora* chromosomes are to specialised metabolism. In line with other actinomycetes, the *Micromonospora* in our sample possessed large chromosomes that were rich in BGCs. What was surprising, however, was the variability in how much ‘genomic real estate’ the genus commits to secondary metabolism, ranging from 3 to 22% of their chromosome. This contrasts with closely related genera, such as *Salinispora*, which devotes ~10% of their genome to specialised metabolism [20]. There was only a weak correlation between both the number of BGCs and genome size and likewise for the percentage of chromosomes that encoded BGCs – this was unsurprising, as there were likely to be other factors at play driving chromosome expansion in *Micromonospora* [53].

Having established this, we next questioned whether BGCs are uniformly distributed across the chromosome or not. In *Streptomyces*, for example, BGCs are predominantly found in the telomeres of the organisms’ linear chromosomes [3]. The BGCs of our *Micromonospora* were distributed across two loci – the ori-distal region contained a rich and diverse set of BGCs, analogous to streptomycetes with linear chromosomes. This was different, however, to the fixation of ori-proximal clusters. We observed that the location of BGCs was partially driven by the class of molecule encoded by that BGC. Type I and II polyketides, nonribosomal peptides and siderophores (the majority of which was desferrioxamine) were found in the ori-distal region. It is suggested that it is the linear nature of streptomycete chromosomes that leads to hybrid replicons and drives genome plasticity and BGC diversity [34] within the taxa. For example, a single recombination event with, say, an incoming linear conjugative plasmid and the chromosome may generate two functional hybrid linear replicons in *Streptomyces*. Our data indicate that, although the generation and resolution of circular hybrid replicons in *Micromonospora* would require at least two recombination events, this genus displays a similar pattern of BGC location around the chromosome terminus as streptomycetes do around the chromosome ends. This challenges the dogma that it is the linear nature of streptomycete chromosomes and concurrent susceptibility to double-strand breaks and recombination, which generates the prodigious biochemical productivity of this genus.

The BiG-SCAPE-generated network of gene cluster families present in *Micromonospora* showed that homologous BGCs are syntenic within the genus (or reverse syntenic in strains with reversed origin islands), which confirms that synteny is maintained through vertical inheritance, even in small GCFs. Previous work has explored the genus-level distribution of BGCs in *Amycolatopsis* [35] and *Salinispora* [54], and this work builds on it by introducing evidence of a core set of BGCs in *Micromonospora*, as well as providing evidence that the nature of the BGC partly determines its fixation into the core set. Further comparison of BGC distribution in other genera of *Actinomycetes*, as well as successful natural producers in other bacterial families, will further shore up our understanding of BGC evolution and the factors driving the incorporation of the BGC and its cognate natural product(s) into an organism’s core suite. Contrary to the core, BGCs are the diverse set present in the ori-distal region of the *Micromonospora* chromosome. This was illustrated by the large number of singleton gene cluster families present in the *oriC*-distal region.

We demonstrated that when split into two regions – the *oriC*-proximal region that describes the chromosome half on either side of the chromosome and the ori-distal region that describes the other half – the ori-distal region consistently possesses a greater diversity of the BGC content. Although not organisms per se, these ecological measurements are proxies for entropy, indicating how difficult it is to predict a sample from a population [55], and so they were appropriate for us to employ, treating different regions as analogous to habitats occupied by BGCs. It could be argued that bisection of the chromosome is a crude way of dividing it, missing the nuance of different genomic islands; however, despite the crudeness, we were able to detect a difference in populations between the two regions.

The question stands: What is the driving factor in the fixation of BGCs in *Micromonospora* chromosomes and the partitioning of BGCs into different populations? The fixed clusters orbited the origin of replication and encoded functions such as compatible solute production [6] and pigment development [49]. NAGGN-type clusters are responsible for the biosynthesis of the compatible solute NAGGN, a dipeptide derived from two units of glutamine. NAGGN is overwhelmingly represented amongst members of the Gram-negative *Pseudomonas* and *Sinorhizobium*, as well as members of the *Micromonosporacea* such as *Salinispora*. This raises the possibility of an ancient horizontal gene transfer event mediating the acquisition of NAGGN clusters. In terpene class BGCs, which were distributed across the chromosome, disruption of the ori-proximal BGCs has been shown to have the greatest downstream impact on pigmentation [49].

What stands out about ori-proximal clusters is that the molecules they synthesize protect against environmental stressors – such as the NAGGN clusters, which protect against desiccation, or pigmentation involved terpene clusters, which protect against UV radiation. This may partially explain the differences between the two populations, and it is easy to hypothesize that organisms that form quiescent spores as part of their life cycle stand to benefit from being able to weather harsh abiotic factors. The accumulation and loss of ori-distal BGCs may then reflect transient usefulness in the evolutionary history of their hosts.

BGCs are constantly evolving genetic entities [33]. Their sheer size and energetic costs of maintenance represent a considerable investment to the organisms that host them. Through the small molecules, they encode and generate fitness benefits to this host. They also contribute to the diversification of their hosts to the point where differences in BGC content between two related organisms can predict interstrain antagonism [28]. Their maintenance depends on occupying a niche within that organism – a function that the molecule they encode fulfils. This has been demonstrated in siderophores in *Salinispora* where some strains have independently lost desferrioxamine biosynthesis in favour of salinichelins [56]. Therefore, BGCs must be under extraordinary selection pressure to maintain their existence. By migrating to the core of the *Micromonospora* chromosome, sharing space with essential genes in the chromosome [57], the core BGC suite in our organisms has become incorporated into the conserved core of the organisms, which implies protection against deletions. This strategy is not guaranteed to preserve the BGC, however, as shown by the introduction of core thiopeptide biosynthetic enzymes into the T3PKS cluster of *M. echinofusca*.

What was conspicuous in the comparison between the BGCs of linear chromosomes and those of the single-contig *Micromonospora* analysed here was the absence of BGCs at the chromosomal equator – halfway between the origin of replication and the ori-distal pole. Two hypotheses may explain this: first, BGC incorporation into the ori-proximal region from the ori-distal region is rare and happens rapidly, with useful BGCs spending little, if any, time at the chromosomal equator. Another hypothesis is that incorporating BGCs into the equator is selected against, thus explaining the absence.

The hyper-variable ori-distal region of the chromosomes seems to be the most likely site of BGC insertion into the chromosome, with BGCs migrating to the core over evolutionary history. An analogous phenomenon has been proposed in *Streptomyces* [33]. It is easy to visualize how a streptomycete linear chromosome permits the replacement of a chromosomal end with that of a linear plasmid by a single recombination event. However, it is less easy to reconcile this with circular *Micromonospora* chromosomes that would require more than one recombination to retain a circular chromosome architecture.

From the outset, we set out to answer three questions: (1) Is there conserved chromosome architecture in *Micromonospora*? (2) Are the BGCs of *Micromonospora* conserved within the genus? (3) Does chromosome architecture play a role in the conservation of BGCs in *Micromonospora*? For the first, we have shown that *Micromonospora* do possess a conserved architecture. This takes the form of an ori-proximal core, with a hyper-variable region at the opposite pole. This pole is likely where termination of DNA replication occurs – it is where strand switching between gene density occurs. For the second question, we have shown that some BGCs within the genus are conserved, namely, terpene, NAGGN and T3PKS clusters. This led us to the answer the third question, which is yes – chromosome architecture does impact the conservation of BGCs and the core suite of BGCs exists as highly syntenic members of the larger *Micromonospora* chromosomal core. The hyper-variable region is populated by a diverse suite of BGCs, implying a high turnover of these clusters. What remains is a question of what exactly drives the discrimination between the core BGCs of the genus and other genes, and how they migrate towards the chromosomal core.

## supplementary material

10.1099/mgen.0.001313 Uncited Fig. S1.

10.1099/mgen.0.001313 Uncited Supplementary Material 1.

## Abbreviations

ANI: average nucleotide identity
BGC: biosynthetic gene cluster
GCF: gene cluster family
NAGGN: N-acetylglutaminylglutamine amide
NRPS: nonribosomal peptide synthase
PKS: polyketide synthase
