# Supplementary material for: Chromosome architecture as a determinant for biosynthetic diversity in Micromonospora
Source: Microb Genom. 2024 Nov 5;10(11):001313. doi: 10.1099/mgen.0.001313 (PMC11537254; doi:10.1099/mgen.0.001313)
Supplement: Uncited Fig. S1. [file mgen-10-01313-s001.pdf]

Supporting Figures

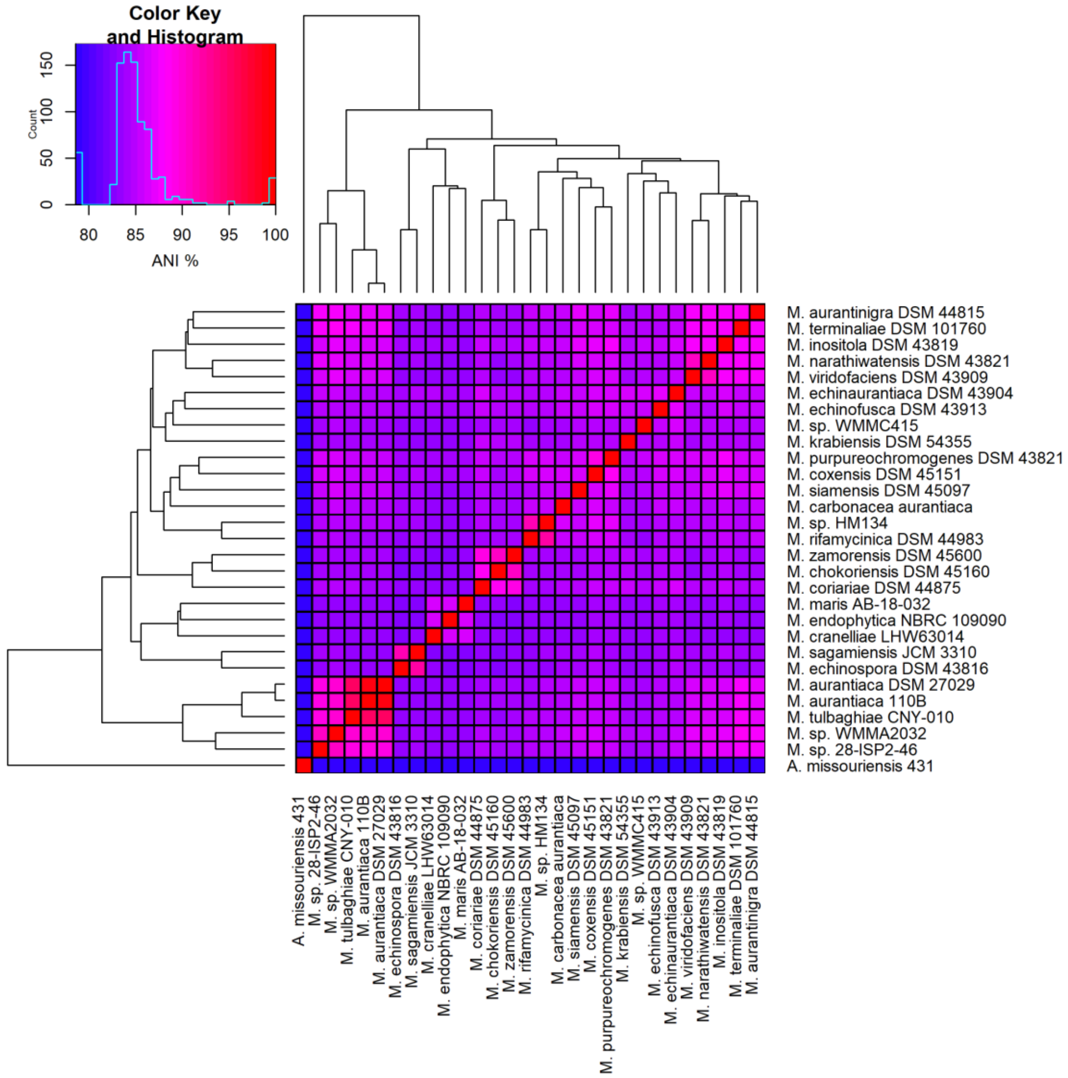

Figure S1 ANI of well assembled *Micromonospora*. Average nucleotide identity was calculated using the FastANI algorithm, and used to inform organisation of alignments presented in Figure 1

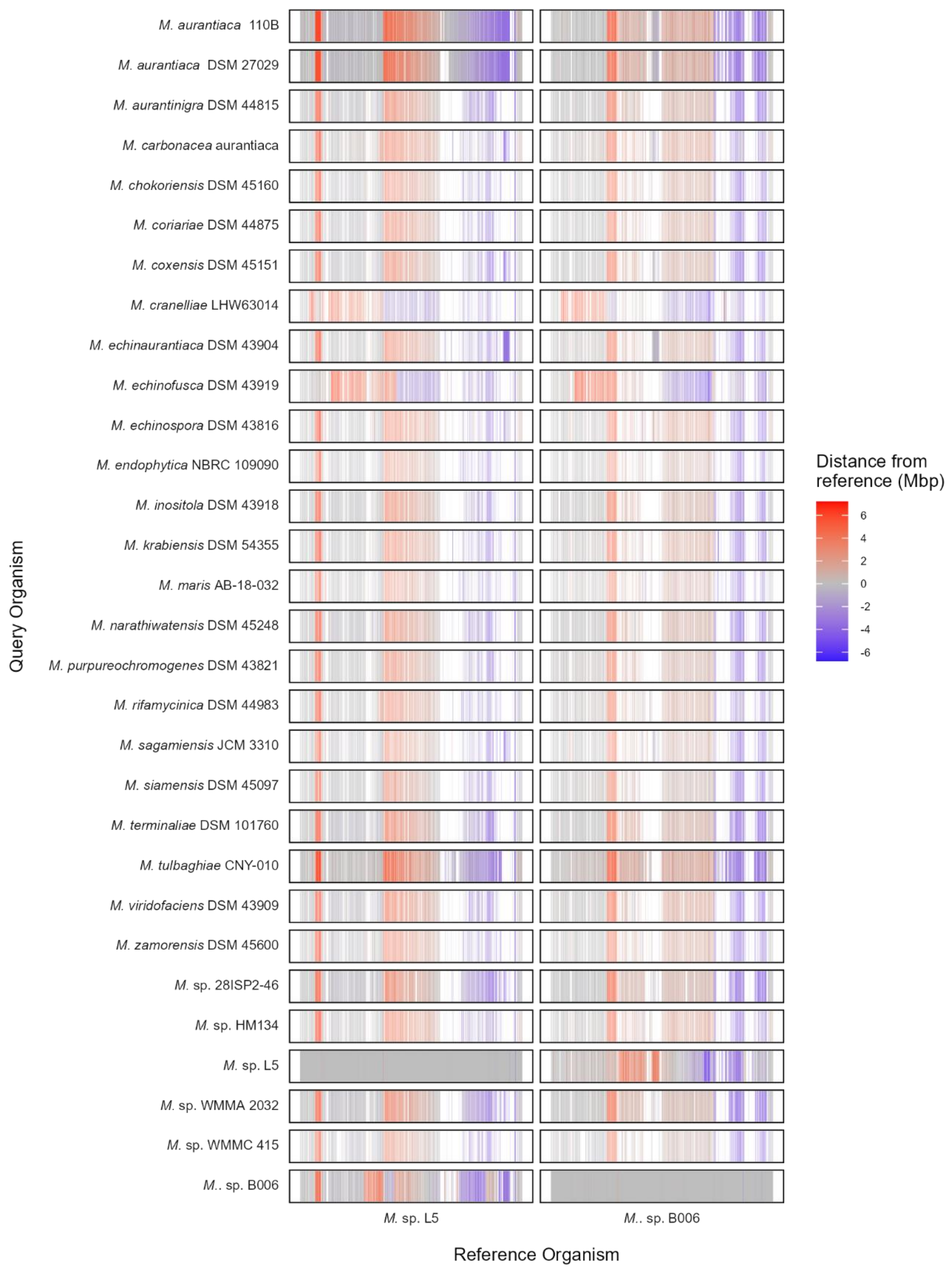

Figure S2. Justification for exclusion of *M. sp.* L5 and *M. sp.* B006. *M.* sp. L5 contains a large inversion which we believe to be biologically implausible, as it would bring the site of DNA replication termination close to the origin of replication if true. *M. sp.* B006 contains a number of spurious rearrangements. Hence, we chose to exclude these two assemblies.

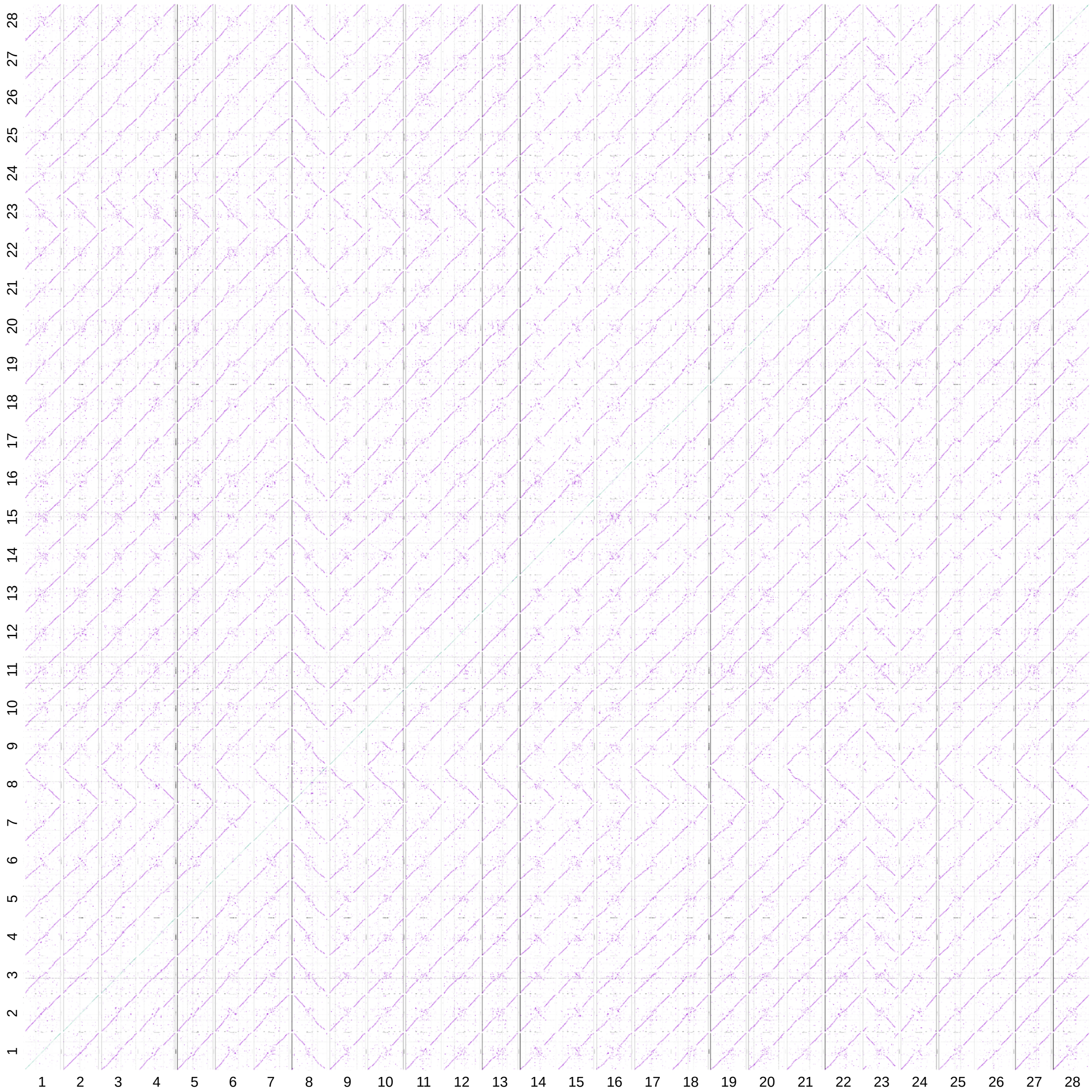

Figure S3. Pairwise nucmer alignments. Dotplots of pairwise nucmer alignments of all 28 strains analysed in this work. The numbers on the bottom left and right of the panel represent strains, as follows.

1: *M.* sp. 28-ISP2-46; 2: *M.* sp. WMMA2032; 3: *M. tulbaghiae* CNY-010; 4: *M. aurantiaca* 110B; 5: *M. aurantiaca* DSM 27029; 6: *M. echinospora* DSM 43816; 7: *M. sagamiensis* JCM 3310; 8: *M. cranelliae* LHW63014; 9: *M. endophytica* NBRC 109090; 10: *M. maris* AB-18-032; 11: *M. coriariae* DSM 44875; 12: *M. chokoriensis* DSM 45160; 13: *M. zamorensis* DSM 45600; 14: *M. rifamycinica* DSM 44983; 15: *M.* sp. HM134; 16: *M. carbonaceae* aurantiaca; 17: *M. siamensis* DSM 45067; 18: *M. coxensis* DSM 45151; 19: *M. purpureochromogenes* DSM 43821; 20: *M. krabiensis* DSM 54355; 21: *M. sp.* WMMC45; 22: *M. echinofusca* DSM 43913; 23: *M. echinaurantiaca* DSM 43904; 24: *M. viridofaciens* DSM 43909; 25: *M. narathiwatensis* DSM 43821; 26: *M. inositola* DSM 43819; 27: *M. terminaliae* DSM 101760; 28: *M. aurantinigra* DSM 44815

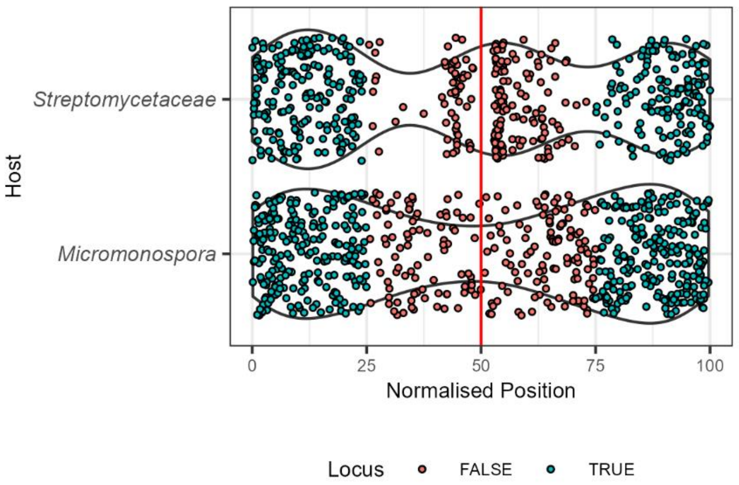

Figure S4. Comparison of BGC distribution in linear and circular chromosomes. Violin plots of BGC distribution in *Streptomycetacea* (19 complete *Streptomyces* chromosomes and 1 complete *Kitasatospora* chromosome) and *Micromonospora* with the *Micromonospora* origin of replication at position 50. *Micromonospora* are distinct by having an *oriC*-proximal suite of BGCs that streptomycetes lack.

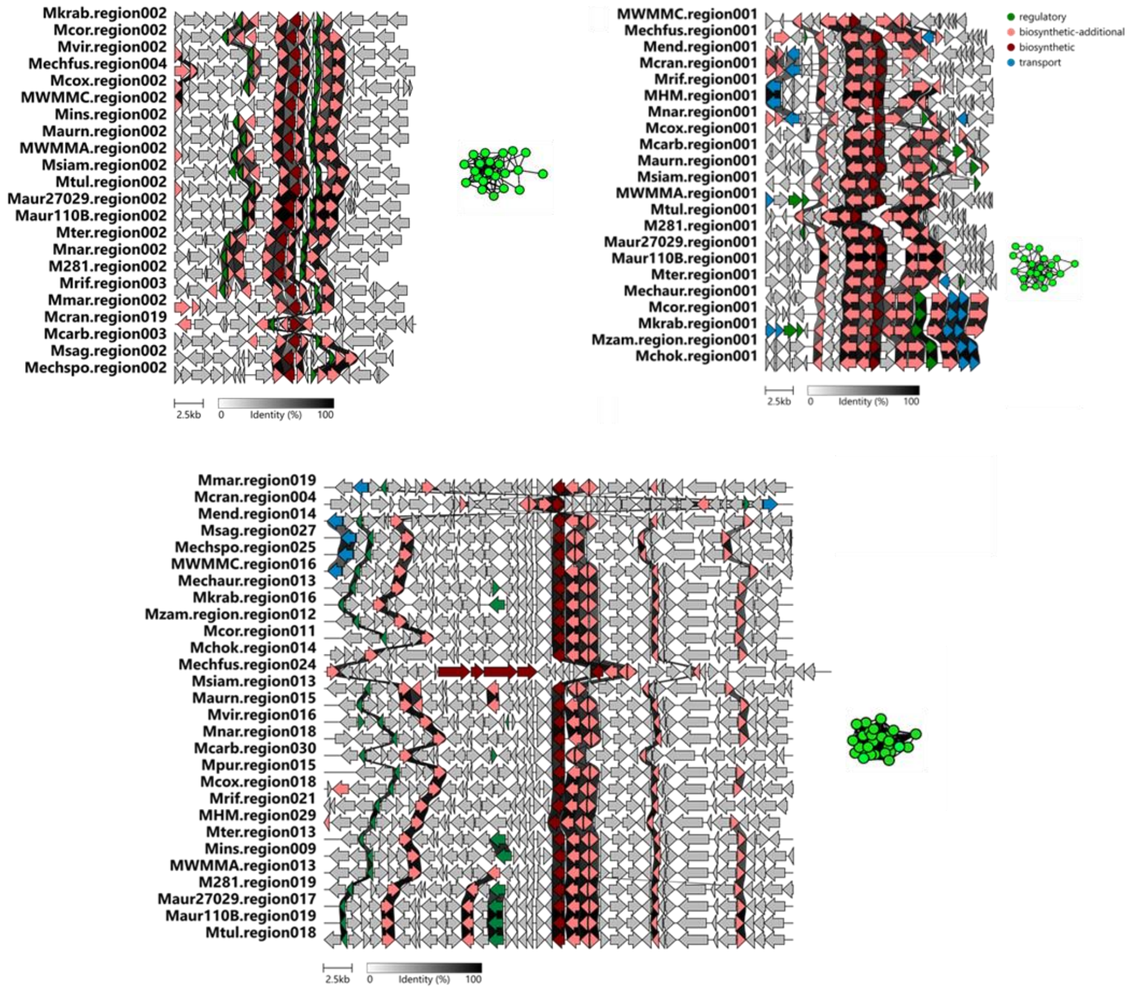

Figure S5. Clinker alignments of conserved, syntenous BGCs. Key biosynthetic enzymes, as predicted by antiSMASH, are annotated.
