# Supplementary material for: Chromosome architecture as a determinant for biosynthetic diversity in Micromonospora
Source: Microb Genom. 2024 Nov 5;10(11):001313. doi: 10.1099/mgen.0.001313 (PMC11537254; doi:10.1099/mgen.0.001313)
Supplement: Uncited Supplementary Material 1. [file mgen-10-01313-s002.pdf]

Organism	Accession	Assembly Level	Included
M. aurantiaca DSM 27029	GCA_000145235.1	Complete	Yes
M. carbonaceae aurantiaca	GCA_013389765.1	Complete	Yes
M. maris AB-18-032	GCA_000204155.1	Complete	Yes
M. cranelliae LHW63014	GCA_014764405.1	Complete	Yes
Micromonospora terminaliae DSM 101760	GCA_009671205.1	Complete	Yes
M. sagamiensis JCM 3310	GCA_014680085.1	Complete	Yes
M. aurantiaca 110B	GCA_003351365.1	Complete	Yes
M. endophytica NBRC 109090	GCA_018326245.1	Complete	Yes
Micromonospora sp. HM134	GCA_007833915.1	Complete	Yes
Micromonospora sp. B006	GCA_003408515.1	Complete	No
Micromonospora sp. L5	GCA_000177655.2	Complete	No
M. echinospora DSM 43816	GCA_900091495.1	Chromosome	Yes
M. rifamycinica DSM 44983	GCA_900090265.1	Chromosome	Yes
M. echinofusca DSM 43913	GCA_900091445.1	Chromosome	Yes
M. aurantinigra DSM 44815	GCA_900089595.1	Chromosome	Yes
M. purpureochromogenes DSM 43812	GCA_900091515.1	Chromosome	Yes
M. echinaurantiaca DSM 43904	GCA_900090235.1	Chromosome	Yes
M. zamorensis DSM 45600	GCA_900090275.1	Chromosome	Yes
M. krabiensis DSM 45344	GCA_900091425.1	Chromosome	Yes
M. viridifaciens DSM 43909	GCA_900091545.1	Chromosome	Yes

M. coriariae DSM 44875	GCA_900091455.1	Chromosome	Yes
M. chokoriensis DSM 45160	GCA_900091505.1	Chromosome	Yes
M. coxensis DSM 45161	GCA_900090295.1	Chromosome	Yes
M. inositola DSM 43819	GCA_900090285.1	Chromosome	Yes
Micromonospora sp. 28ISP2-46	GCA_013694245.1	Chromosome	Yes
M. narathiwatensis DSM 45248	GCA_900089605.1	Chromosome	Yes
M. siamensis DSM 45907	GCA_900090305.1	Chromosome	Yes
Micromonospora sp. WMMA2032	GCA_002688545.1	Chromosome	Yes
Micromonospora sp. WMMC415	GCA_009707425.1	Chromosome	Yes
M. tulbaghiae CNY-010	GCA_003612775.1	Complete	Yes
